# Be or Not to Be With ChatGPT?

**DOI:** 10.7759/cureus.48366

**Published:** 2023-11-06

**Authors:** Aynur Aliyeva, Elif Sari

**Affiliations:** 1 Department of Otolaryngology - Head and Neck Surgery, Cincinnati Children's Hospital Medical Center, Cincinnati, USA; 2 Department of Otorhinolaryngology - Head and Neck Surgery, Istanbul Aydın University VM Medical Park Florya Hospital, Istanbul, TUR

**Keywords:** artificial intelligence, future in medicine, research, scientific manuscript, chatgpt

## Abstract

In the ever-evolving realm of scientific research, this letter underscores the vital role of ChatGPT as an invaluable ally in manuscript creation, focusing on its remarkable grammar and spelling error correction capabilities. Furthermore, it highlights ChatGPT's efficacy in expediting the manuscript preparation process by streamlining the collection and highlighting critical scientific information. By elucidating the aim of this letter and the multifaceted benefits of ChatGPT, we aspire to illuminate the path toward a future where scientific writing achieves unparalleled efficiency and precision.

## Editorial

To the Editor,

In the rapidly advancing scientific landscape, tools that aid in the streamlined creation of research papers are not just luxuries but necessities. ChatGPT has emerged as a stellar auxiliary tool in this context, aiming to enhance the quality and efficiency of scientific manuscript writing [1]. This letter outlines the indispensable roles played by ChatGPT in facilitating the production of high-quality scientific manuscripts, focusing on their grammatical prowess and ability to sift through and analyze voluminous scientific data.

Grammar and spelling error correction: ChatGPT has exhibited a remarkable capacity for grammar and spelling error correction, essentially serving as a vigilant editor that enhances the linguistic quality of the manuscript. It effectively eradicates errors that could potentially mar the readability and credibility of a research document, thereby acting as a reliable first line of defense against linguistic imperfections [2].

Facilitating the collection and highlighting of scientific information: In the cutthroat environment where timely publication can be crucial, ChatGPT serves scientists by accelerating the manuscript preparation process. It enables a swift review of the general results found in various articles, highlighting pivotal data for the discussion section, thereby augmenting scientific discourse's overall quality and depth [3].

ChatGPT shines in helping researchers to collate and focus on pertinent scientific data drawn from a plethora of sources, guiding them in formulating well-grounded arguments and discussions. This ensures a higher quality manuscript that can be completed in a fraction of the time, offering researchers the luxury of focusing more on analysis rather than the arduous data collection task [4].

Limitations of ChatGPT in scientific writing: While ChatGPT can provide general insights and generate text based on its last training data, it is not ideal for sourcing the latest citations or highly specialized sources. Its knowledge is limited to the cut-off date of its last training set, and it lacks real-time access to current databases or journals. Thus, researchers must cross-check and source citations from credible and up-to-date platforms. ChatGPT's expertise might not cover the depth and breadth required in highly specialized or niche fields. While it can generate text based on its training, it does not replace domain experts or specialized databases. One potential risk is the oversimplification of complex topics. ChatGPT aims to generate coherent and contextually relevant responses, but there might be cases where the generated content lacks the depth or nuance required for scientific discourse. While ChatGPT can be a useful tool for initial drafts or brainstorming, researchers must critically evaluate and verify the information generated. It is essential to uphold the rigorous standards of scientific writing and ensure the accuracy and credibility of the content [5].

The future with ChatGPT: Envisioning the roadmap ahead, ChatGPT stands as a frontier tool poised to redefine the terrain of scientific paper writing. It harbors the potential to foster collaborations, bridge gaps, and enhance research manuscripts' comprehensiveness.

As we contemplate the question, "to be or not to be with ChatGPT," it becomes clear that embracing this tool could mean stepping into an era of heightened efficiency and precision in scientific writing. It demands a balanced approach, where we utilize ChatGPT to its fullest while retaining a critical approach to verify and substantiate the information it provides, thus upholding the principles of scientific rigor [5].

By merging the advanced capabilities of ChatGPT with human ingenuity, we can foster a future where scientific manuscripts are not just technically sound but are enriched with depth, focusing on groundbreaking discoveries in science.

With the aid of ChatGPT, scientific research papers can achieve a harmonious marriage of quality and efficiency, promising a revolutionized approach to manuscript writing. The tool ensures grammatical correctness and acts as a researcher's assistant, aiding data collection and its intelligent highlight in discussions, fostering ground for deeper and more nuanced scientific analysis.

Yours faithfully
